# The Temperature Effect on the Electrochemical Performance of Sulfur-Doped LiMn_2_O_4_ in Li-Ion Cells

**DOI:** 10.3390/nano9121722

**Published:** 2019-12-05

**Authors:** Marcelina Kubicka, Monika Bakierska, Michał Świętosławski, Krystian Chudzik, Marcin Molenda

**Affiliations:** Faculty of Chemistry, Jagiellonian University, Gronostajowa 2, 30-387 Krakow, Poland; lis@chemia.uj.edu.pl (M.K.); monika.bakierska@uj.edu.pl (M.B.); krystian.chudzik@doctoral.uj.edu.pl (K.C.)

**Keywords:** Li-ion battery, spinel-based cathode material, temperature dependence, harsh conditions

## Abstract

The application of modified spinel materials in commercial systems relates to the verification of their parameters under different conditions. Hence, in this study, the influence of temperature on the electrochemical behavior of sulfur-doped spinel (LiMn_2_O_3.97_S_0.03_), with reference to stoichiometric spinel (LiMn_2_O_4_), was investigated. The electrochemical characteristics of Li-ion cells based on the fabricated spinels were investigated using galvanostatic charge–discharge tests (GCDT), cyclic voltammetry (CV), and electrochemical impedance spectroscopy (EIS). The results show that introducing sulfur into the spinel structure improves the electrochemical properties at each of the studied temperatures (3, 22 and 61 °C). LiMn_2_O_3.97_S_0.03_ exhibits higher specific capacities, enhanced diffusivity and charge–discharge rates (particularly at low temperatures), and much better cycling stability, regardless of the testing conditions. Our research proves that an S-doping system is a better alternative to LiMn_2_O_4_ in the context of working parameters, while maintaining environmental friendliness and low manufacturing costs.

## 1. Introduction

The rapid progress of industry is one of the reasons for the incessantly increasing worldwide demand for energy storage systems [1,2]. Among all currently available technologies, lithium-ion batteries (LIBs) appear to be the most successful, due to their high energy density, good performance and long cycle life [3,4]. Considering the rapid growth of the application of LIBs in the global markets, battery development in the 21st century needs to be targeted on green production processes as well as sustainability with respect to operational parameters and safety issues [4,5,6].

Nowadays, of various kinds of cathode materials for rechargeable lithium-ion batteries, lithium manganese oxide spinel (LiMn_2_O_4_, LMO) is extensively investigated in order to meet industrial, environmental and functional requirements [7,8]. The attractiveness of LiMn_2_O_4_ is connected with its specific capacity, high operating voltage, low-cost ingredients and, importantly, its environmentally friendliness [9,10]. However, LMO is unstable in Li-ion cells. Near room temperature, one can observe a reversible adverse phase transition, which is related to the Jahn–Teller distortion of high-spin Mn^3+^ ions. Furthermore, stoichiometric spinel has limited stability towards liquid electrolytes, which results in its decomposition and partial dissolution. These phenomena lead to a decrease in capacity during the charge–discharge cycles [11,12,13]. One of the possible strategies to deal with the aforementioned problems is the modification of LMO composition by the partial or synergic substitution of the cationic/anionic sublattices [14,15]. In our previous studies on spinel materials, it was shown that doping LiMn_2_O_4_ with sulfur improves the material, structural and electrochemical stability [16,17,18,19].

However, the application of new, modified spinel materials in commercial Li-ion cells is associated with the verification of their properties under different conditions. It is commonly known that although normal, steady exploitation ensures the optimal parameters of the battery, intensive usage under harsh conditions may significantly affect its capacity and overall efficiency [20,21,22]. Therefore, in the present work, the temperature effect on the electrochemical performance of LiMn_2_O_3.97_S_0.03_ (LMOS_0.03_), with high commercial potential, is investigated.

## 2. Materials and Methods 

The spinel materials, LiMn_2_O_4_ (LMO) and LiMn_2_O_3.97_S_0.03_ (LMOS_0.03_), were obtained by a xerogel type, water-based, sol–gel method followed by a two-step calcination process, first at 300 °C for 24 h (heating rate of 1 °C min^−1^), and then at 650 °C (heating rate of 5 °C min^−1^) with quench after 6 h (described in detail previously by our group [16,17,18,19]). To introduce sulfur into the spinel structure, with a simultaneous 10% excess of lithium, the lithium sulfide (Sigma-Aldrich, 99.98%) was added to the flask before the precipitation.

The crystal structure of the spinel materials was analyzed using X-ray powder diffraction (XRD), using a Bruker D2 PHASER diffractometer (Billerica, MA, USA) with a Cu lamp K_α1_ radiation (λ = 0.154184 nm) between 10° and 80° 2θ, with a step of 0.02°. To determine the presence of the phase transition, differential scanning calorimetry (DSC) experiments were carried out on a Mettler-Toledo 821 e instrument equipped with intracooler Haake (Mettler-Toledo, Columbus, OH, USA) in the temperature range of −20° to +50 °C with a heating and cooling rate equal to 10 °C min^−1^ under constant flow of argon. The sulfur content in the LMOS sample was examined by elemental analysis (EA) (micro analyzer Vario MICRO cube coupled with microbalance, Elementar).

In order to investigate the electrochemical performance, galvanostatic charge–discharge tests (GCDT) were conducted using the ATLAS 1361 MPG&T multichannel battery tester (ATLAS–SOLLICH, Rębiechowo, Poland) in 3.0–4.5 V potential range, under different current loads, at three selected temperatures: 3, 22 and 61 °C. The working electrodes were prepared by grinding 80 wt.% of active material, 10 wt.% of Carbon Black- Super P Conductive (Alfa Aesar, >99%) and 10 wt.% of poly (vinylidene fluoride) (Sigma Aldrich) with N-methyl−2-pyrrolidone as a solvent (Sigma Aldrich, ≤99.5%) in a ball mill. Then, the slurry was coated on aluminum foil and dried at 120 °C in a vacuum dryer for 24 h. The cells were assembled in an argon-filled glove box (MBraun glove box, Garching, Germany) with a trilaminate of polypropylene/polyethylene/polypropylene film (Celgard 2325) (Celgard LLC, Charlotte, NC, USA) and two porous glass microfiber filters (Whatman GF/F) (Sigma Aldrich, Saint Louis, MO, USA) as separators, 1 M LiPF_6_ in a mixed-solvent of ethylene carbonate and diethyl carbonate EC/DEC (50/50 v/v, Sigma-Aldrich, battery grade) as electrolyte as well as lithium foil (Sigma Aldrich, 99.9%) as an anode material. The electrochemical impedance spectroscopy (EIS) and cyclic voltammetry (CV) were carried out on the potentiostat/galvanostat (AUTOLAB PGSTAT302 N/FRA2, Metrohm Autolab, Utrecht, The Netherlands). The EIS measurements were performed at 3.75 V voltage of the cells by applying 0.1 V amplitude, in the frequency range of 100 kHz to 0.03 Hz. The impedance data were then fitted using Nova software (version 1.11, Metrohm Autolab, Utrecht, The Netherlands). The CV scans were conducted at different scan rates of 0.05, 0.075, 0.1, 0.25, and 0.5 mV s^−1^, in the potential range of 3.0 to 4.5 V (vs. Li/Li^+^) starting from an open circuit voltage (OCV).

## 3. Results and Discussion

**Figure 1:**
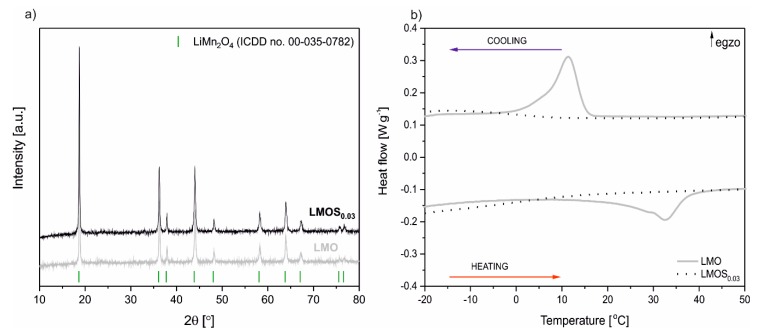
(**a**) X-ray diffraction (XRD) patterns of LiMn_2_O_4_ as well as LiMn_2_O_3.97_S_0.03_; (**b**) the differential scanning calorimetry (DSC) curves of the spinel materials.

Figure 1a presents the XRD diffraction patterns of LiMn_2_O_4_ (LMO) as well as LiMn_2_O_3.97_S_0.03_ (LMOS_0.03_) powders. All the diffraction lines can be attributed to the single phase cubic LMO spinel structure with the Fd–3 m space group (ICDD No. 00–035–0782). The lattice parameter of the LiMn_2_O_3.97_S_0.03_ (0.8212 nm) is slightly higher than that of the stoichiometric spinel (0.8181 nm). This results from the dominant lattice expansion, an effect related to sulfur substitution for oxygen in the spinel structure [16,17]. The average crystallite size calculated using the Scherrer formula is equal to 42 nm and 48 nm for LMOS_0.03_ and LMO, respectively. The results of the DSC measurements of the obtained spinels are illustrated in Figure 1b. The LMO DSC heat–flow curve has visible characteristic heat effects associated with the first order phase transition of the spinel from a cubic to an orthorhombic structure. For doped LMOS_0.03_, the sulfur stabilizes the spinel structure, the phase transition is suppressed, and heat effects are significantly reduced. These results are consistent with our previous studies [15,16,17,18,19,23], where we showed that sulfur substitution into the oxygen sublattice of lithium manganese oxide spinel can be successfully implemented in sol–gel synthesis. The presence of sulfur was also confirmed using an elemental analysis and was estimated as 0.445 wt.%, which gives the exact composition of LiMn_2_O_3.97_S_0.03_.

**Figure 2:**
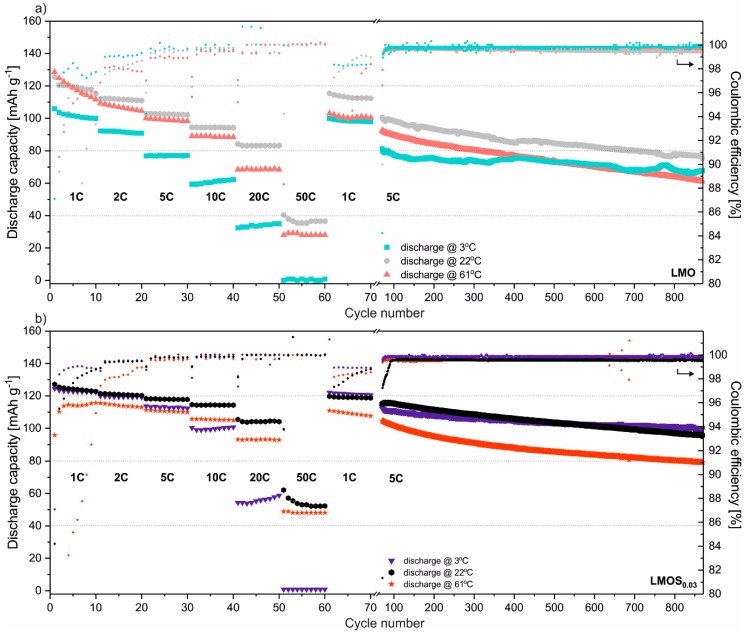
Galvanostatic charge–discharge studies of (**a**) Li/LMO and (**b**) Li/LMOS_0.03_ under various conditions.

In Figure 2, galvanostatic charge–discharge tests of stoichiometric LMO and sulfur-doped LMOS_0.03_ spinel-based cells are presented. All the studied cells were tested performing 10 cycles in sequence using currents equivalent of 1C, 2C, 5C, 10C, 20C, 50C, 1C and 800 cycles under 5C current load. The typical mass loading of active material was established to 1.08 mg cm^−2^, and 0.99 mg cm^−2^ for LMO- and LMOS_0.03_-based electrodes, respectively. Cells were tested in three different temperatures of 3, 22 and 61 °C, representing the most common operating range of Li-ion storage systems. What is apparent from these results is the significant influence of sulfur on the electrochemical properties of spinel at low temperatures and under high currents. Stoichiometric LMO, cycled at 3 °C, in comparison to studies at 22 °C, delivers ca. 15% less discharge capacity under 1C, ca. 25% less under 5C, and up to 60% less when fast-discharged using a 20C current rate. This effect results from suppressed lithium ion mobility in the spinel structure at low temperatures. Sulfur doping causes the slight expansion of the structure, whilst at the same time increasing the apparent diffusion coefficient (based on CV studies presented further in the text), and enabling faster Li^+^ migration in the cathode. This effect is observable regardless of cell operating temperatures, but is most obvious in samples studied at low temperatures under high current loads. For sulfur-doped LMOS_0.03_, the discharge capacity remains almost the same for cells cycled at 3 and 22 °C when using current rates up to 2C (ca. 120 mAh g^−1^). Raising the current load to 5C results in a ca. 5% capacity decrease (113 mAh g^−1^ at 3 °C). The sulfur-doping effect is tangible even under extreme 50C discharge rates, when the LMOS_0.03_ cell delivers ca. 50% more capacity than stoichiometric LMO (cells at 22 °C). The S stabilization effect is not as obvious at an elevated temperature (61 °C). Sulfur-doped material is still more stable, with a higher columbic efficiency and a higher capacity than the undoped material, but the cell performance of both spinels is clearly worse than when working at room temperature. A very low columbic efficiency in initial cycles (up to 20th) suggests the simultaneous occurrence of side reactions in the cells. The Mn dissolution from the spinel structure strongly depends on the concentration of acid species in the electrolyte. Elevated temperatures accelerate the decomposition of LiPF_6_ salt with the generation of acidic HF (PF_5_ reaction with water residues), which causes a disproportionation reaction (2 Mn^3+^ → Mn^4+^ + Mn^2+^) at the particle’s surface [24,25]. The overall deterioration of electrochemical performance results not only from loss of active material, but also from the deposition of the spinel, and electrolyte decomposition products such as LiF, MnF_2_, δ-MnO_2_, and Mn-P complexes on the surface of the electrode [26]. The presented results show that even though sulfur doping of the spinel structure does not protect material from the attack of acidic species generated in electrolyte, the LMOS_0.03_ is much more stable, even at elevated temperatures. The capacity retention, calculated between the 21st and 870th cycles, is 62% for LMO and 73% for LMOS_0.03_.

**Figure 3:**
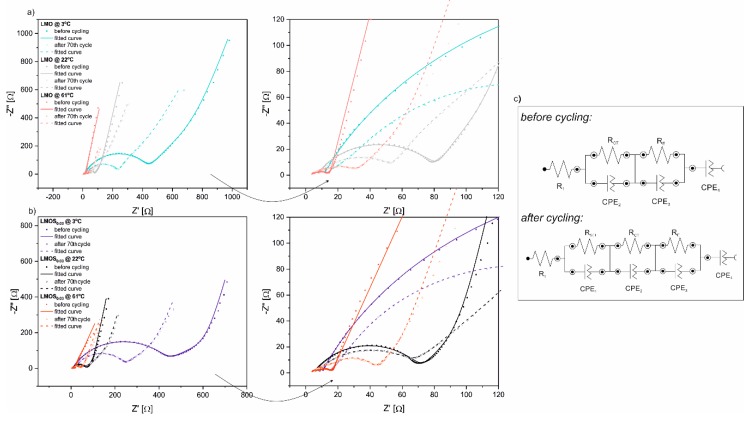
Electrochemical impedance spectra of (**a**) Li/LMO and (**b**) Li/LMOS_0.03_ at 3.75 V before cycling and after the 70th cycle at various temperatures with fitted curves as well as (**c**) the equivalent circuit models.

Figure 3 presents Nyquist plots from EIS measurements, along with close-ups of impedance spectra at the high-to-medium frequency region of Li/LMO (Figure 3a) and Li/LMOS_0.03_ (Figure 3b) half-cells, recorded before cycling and after the 70th cycle at various temperatures. These Nyquist plots reflect familiar electrochemical behavior for compounds of the LiMn_2_O_4_-based spinel group, as already reported and discussed in detail in our previous studies [15,18]. Each curve is composed of three flat semicircles at the high-to-low frequency region, and a sloped straight line at the very low frequency range, corresponding to passivation layer formation, charge transfer reaction, electron properties of the spinel material, and Li^+^-ion diffusion, respectively. Furthermore, the high frequency intercept at the real axis is assigned to the ohmic resistance of the cells. The exceptions are the EIS spectra collected before the galvanostatic charge–discharge tests, which consist only of two depressed semicircles and a tail, as the passivation layer creation occurs during electrochemical performance of the cells, and does not give any contribution to the impedance of the uncycled cells. The impedance data were well fitted with the equivalent circuit models displayed in Figure 3c [27]. In these circuits, particular elements simulate the visible EIS response and refer to the processes mentioned above. Generally, the differences in the parameters of EIS measurements (mainly calculated values of resistors in proposed equivalent circuits) between LMO- and LMOS_0.03_-based cells are negligible (Table S1). The R_1_ values, assigned to the ohmic resistance, are quite small and gradually decrease as the temperature increases for the two investigated cathode materials. A much more pronounced influence of temperature takes place in the case of charge transfer resistance, R_CT_, as the cells measured at 3 °C exhibit higher values (R_CT_ vary from about 440 Ω before cycling to 220 Ω after the 70th cycle for LMO as well as LMOS_0.03_) than those obtained at 61 °C (R_CT_ amounts about 4 Ω before cycling and 25 Ω after 70 cycles for both studied electrodes). In fact, the decrease in charge transfer resistance, together with the temperature increase, contributes to improved kinetics and, consequently, better rate capability, which is partially consistent with the results obtained from charge–discharge tests, discussed above. Nevertheless, it is worth noting that electrochemical tests of cells at 3 °C lead to the decline of R_CT_ resistance after 70 cycles, in comparison to their initial value. Contrastingly, the cells tested at 61 °C demonstrate higher R_CT_ values after cycling. This effect directly results from the deposition of LiF, MnF_2_ and other species at the surface of the electrode. The thick, resistive passivation layer limits lithium diffusion and current flow, resulting in higher cell polarization. In turn, at 22 °C, comparable charge transfer resistances are observed for LMO and LMOS_0.03_ samples before and after cycling (ca. 60 Ω), indicating the stable cycling performance of spinel cathodes at room temperature.

**Figure 4:**
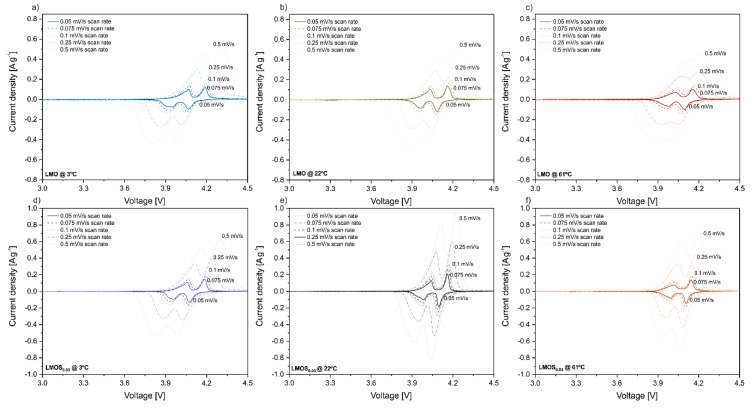
Cyclic voltammograms performed using different scan rates for Li/LMO at (**a**) 3 °C, (**b**) 22 °C and (**c**) 61 °C as well as for Li/LMOS_0.03_ at (**d**) 3 °C, (**e**) 22 °C and (**f**) 61 °C.

In Figure 4, cyclic voltammograms for LMO and LMOS_0.03_ measured at 3, 22 and 61 °C are shown. Both materials in different conditions exhibit a linear relation between peak current density and square root of scan rate (Figures S1–S3 in Supplementary Materials, R^2^ linear fit correlations exceed 0.99). Thus, the apparent diffusion coefficients (D_Li+_) were calculated using the Randles–Sevick Equation (1):

(1) $$I_{p}=0.4463\cdot n^{\frac{3}{2}}\cdot A\cdot F^{\frac{3}{2}}\cdot D^{\frac{1}{2}}\cdot C\cdot v^{\frac{1}{2}}\cdot R^{-\frac{1}{2}}\cdot T^{-\frac{1}{2}}$$

where *I_p_* is the peak current in voltammetric scan, *n* is the number of electrons transferred during the electrochemical reaction (1 for spinel based materials), *A* is the surface of the electrode (1.131 cm^2^ for electrode used in these studies), *F* is the Faraday constant, *C* is the bulk concentration of Li^+^ ions (0.024 mol/cm^3^ for LiMn_2_ O_4_), *ν* is the scan rate, *R* is the gas constant, and *T* is temperature. 

**Figure 5:**
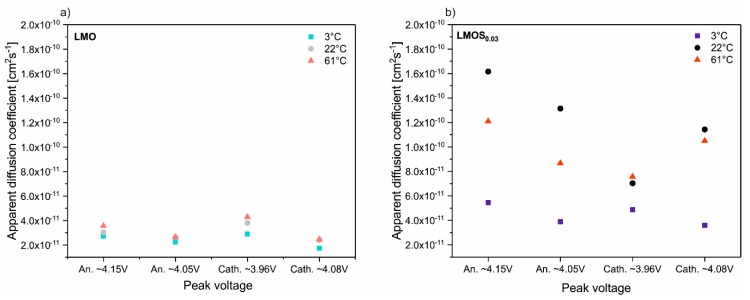
The D_Li+_ calculations for (**a**) Li/LMO and (**b**) Li/LMOS_0.03_ cells at different temperatures.

The D_Li+_ calculations for all the LMO and LMOS_0.03_ peaks presented in CV scans in mentioned temperatures are illustrated in Figure 5 (the average values are collected in Table 1). As can be seen, in the case of LiMn_2_O_4_, the dependence of D_Li+_ on the temperature is minor, nonetheless D_Li+_ slightly increases with the temperature growth. On the other hand, the sulfur-doped material exhibits significant changes of the D_Li+_ with a temperature increase (two-fold increase at 3 °C, four-fold at 22 °C and three-fold at 61 °C in comparison to stoichiometric spinel). These changes are also reflected in the increase in S-doped spinel performance in the galvanostatic charge–discharge tests, that can be attributed to the expansion of the spinel lattice caused by sulfur substitution. What is more, the diffusivity at 61 °C is enhanced, which translates into better rate capability for both materials when compared to 3 °C. However, side reaction rates are also higher when the temperature rises, which is also confirmed by long-term charge–discharge tests, where a major capacity fade at 61 °C is observed for both materials. The effect of the occurrence of side reactions is particularly important for LMOS_0.03_-based cells, where a notable decrease in D_Li+_ is noticed at elevated temperatures.

## 4. Conclusions

The sulfur-doped lithium manganese spinel shows better electrochemical properties under all studied conditions. The presence of S in the structure significantly influences D_Li+_ and charge–discharge rates, especially when operating at low temperatures. Even though S-doping does not seem to protect material from dissolution in the electrolyte when acidic species are present in the system, LMOS_0.03_ is much more stable in long-term cycling, regardless of cell temperature. These studies clearly prove that simple LMO modification can significantly extend the life of the cell and allow better utilization of the material’s properties, which is crucial for the use of this inexpensive and environmentally friendly material in large-scale commercial systems.

## Figures and Tables

**Table 1:** Dependence of the average D_Li+_ on the temperature for stoichiometric and S-doped spinel.

	D_Li+_ [cm^2^ s^−1^] for LMO	D_Li+_ [cm^2^ s^−1^] for LMOS_0.03_
3 °C	2.40∙10^−11^	4.45∙10^−11^
22 °C	2.98∙10^−11^	11.90∙10^−11^
61 °C	3.23∙10^−11^	9.71∙10^−11^

## Supplementary Materials

The following are available online at https://www.mdpi.com/2079-4991/9/12/1722/s1, Table S1: Parameters of EIS measurements for LMO- and LMOS_0.03_-based electrodes, Figure S1: The linear relation between peak current density and square root of scan rate for (**a**) LiMn_2_O_4_ and (**b**) LiMn_2_O_3.97_S_0.03_ at 3 °C, Figure S2: The linear relation between peak current density and square root of scan rate for (**a**) LiMn_2_O_4_ and (**b**) LiMn_2_O_3.97_S_0.03_ at 22 °C, Figure S3: The linear relation between peak current density and square root of scan rate for (**a**) LiMn_2_O_4_ and (**b**) LiMn_2_O_3.97_S_0.03_ at 61 °C.
