# Supplementary material for: The Temperature Effect on the Electrochemical Performance of Sulfur-Doped LiMn2O4 in Li-Ion Cells
Source: Nanomaterials (Basel). 2019 Dec 5;9(12):1722. doi: 10.3390/nano9121722 (PMC6955771; doi:10.3390/nano9121722)
Supplement: Supplementary file 1 [file nanomaterials-09-01722-s001.pdf]

nanomaterials

Supplementary materials

The temperature effect on the electrochemical performance of sulfur doped $LiMn_2O_4$ in Li-ion cells

Marcelina Kubicka [1], Monika Bakierska [1], Michał Świętosławski [1,*], Krystian Chudzik [1] and Marcin Molenda [1,*]

[1] Faculty of Chemistry, Jagiellonian University, Gronostajowa 2, 30-387 Krakow, Poland; lis@chemia.uj.edu.pl (M.K.); monika.bakierska@uj.edu.pl (M.B.); krystian.chudzik@doctoral.uj.edu.pl (K.C.)
* Correspondence: m.swietoslawski@uj.edu.pl (M.Ś); molendam@chemia.uj.edu.pl (M.M.); Tel.: +48-12-6862422 (M.Ś.); +48-12-6862419 (M.M.)

Table S1. Parameters of EIS measurements for LMO and $LMOS_{0.03}$ based electrodes.

		Li/LMO				Li/$LMOS_{0.03}$			
		R_1	R_{SEI}	R_{CT}	R_E	R_1	R_{SEI}	R_{CT}	R_E
**5°C**	before cycling	11	-	443	244	10	-	441	161
	after 70th cycles	12	14	213	106	14	7	227	124
**25°C**	before cycling	7	-	76	26	5	-	67	24
	after 70th cycles	5	3	48	61	5	2	61	116
**60°C**	before cycling	4	-	4	7	4	-	4	8
	after 70th cycles	5	4	26	26	4	15	23	13

R_1, R_{SEI}, R_{CT} and R_E stand for ohmic resistance, solid electrolyte interface (SEI) resistance, charge transfer resistance and electronic resistance, respectively

Linear relation between peak current density and square root of scan rate- for effective lithium ion diffusion coefficients (D_{Li+}) calculations

Both spinel materials in different conditions exhibit linear relation between current and square root of scan rate (R^2 linear fit correlations exceed 0.99).

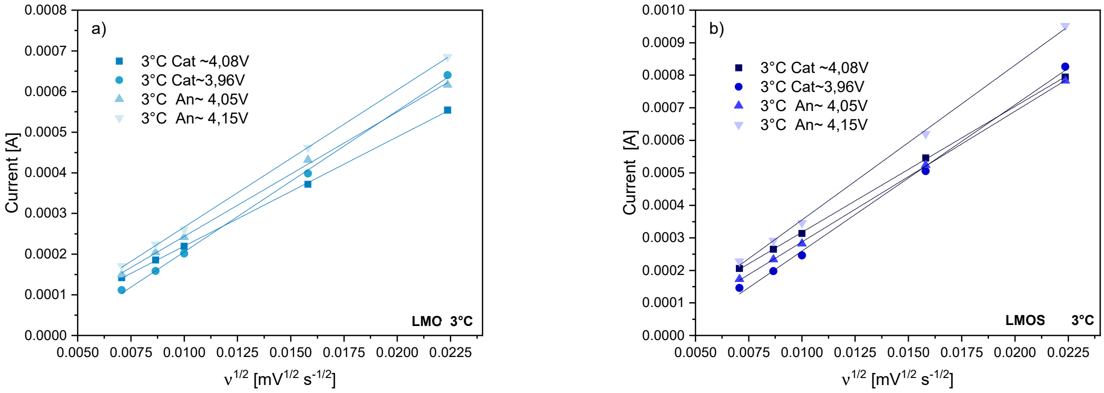

Figure S1. The linear relation between current and square root of scan rate for (**a**) $LiMn_2O_4$ as well as (**b**) $LiMn_2O_{3.97}S_{0.03}$ at 3°C.

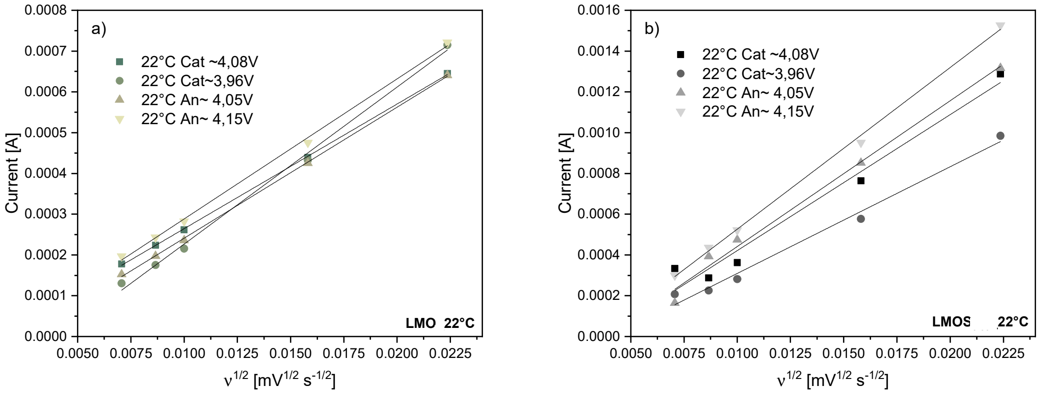

Figure S2. The linear relation between current and square root of scan rate for (**a**) $LiMn_2O_4$ as well as (**b**) $LiMn_2O_{3.97}S_{0.03}$ at 22°C.

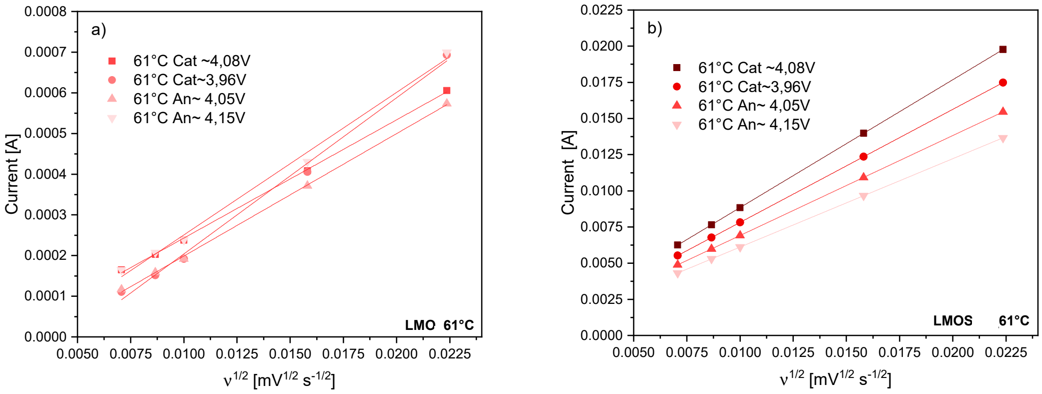

Figure S3. The linear relation between current and square root of scan rate for (**a**) $LiMn_2O_4$ as well as (**b**) $LiMn_2O_{3.97}S_{0.03}$ at 61°C.

© 2019 by the authors. Submitted for possible open access publication under the terms and conditions of the Creative Commons Attribution (CC BY) license (http://creativecommons.org/licenses/by/4.0/).
